# All That Glitters Like Gold Is Not Good: A Rare Constellation of Pericardial Effusion Without Cardiac Tamponade and Biochemical Myopathy in Hypothyroidism

**DOI:** 10.7759/cureus.94970

**Published:** 2025-10-20

**Authors:** Usamah Al-Anbagi, Muayad K Ahmad, Abdulrahman Saad, Tarek Ibrahim, Abdulqadir J Nashwan, Mohamed G Mohamedali

**Affiliations:** 1 Internal Medicine, Hazm Mebaireek General Hospital, Hamad Medical Corporation, Doha, QAT; 2 Internal Medicine, Hamad Medical Corporation, Doha, QAT; 3 Medicine, Ministry of Public Health, Doha, QAT; 4 Pharmacy, Hamad Medical Corporation, Doha, QAT; 5 Nursing and Midwifery Research, Hamad Medical Corporation, Doha, QAT

**Keywords:** case report, endocrine cardiology, pericardial effusion, rhabdomyolysis, severe hypothyroidism

## Abstract

Severe hypothyroidism can present with a wide range of clinical manifestations, often involving multiple organ systems and mimicking primary cardiac or metabolic disorders. We report the case of a 45-year-old man who presented with chest pain, marked bradycardia, and moderate pericardial effusion, along with bicytopenia and rhabdomyolysis. Initial evaluation raised suspicion for acute coronary syndrome; however, laboratory testing revealed markedly elevated thyroid-stimulating hormone and low free thyroxine levels, confirming the diagnosis of severe primary hypothyroidism. The patient demonstrated gradual clinical improvement with levothyroxine therapy, including resolution of bradycardia, pericardial effusion, and biochemical abnormalities. This case highlights the importance of maintaining a high index of suspicion for hypothyroidism in patients presenting with unexplained cardiovascular or neuromuscular symptoms. Early recognition and appropriate thyroid hormone replacement can prevent potentially life-threatening complications and avoid unnecessary invasive interventions.

## Introduction

Hypothyroidism is a common endocrine disorder characterized by insufficient production of thyroid hormones, which play a vital role in regulating metabolic, cardiovascular, and neuromuscular functions [1,2]. While typical symptoms include fatigue, weight gain, cold intolerance, and constipation, severe or untreated hypothyroidism can result in widespread systemic involvement affecting the heart, skeletal muscles, hematologic system, and other organs [1,3].

Cardiovascular manifestations are among the most clinically significant and may include bradycardia, diastolic hypertension, and pericardial effusion, with cardiac tamponade being a rare but potentially fatal complication [4,5]. The pathogenesis of pericardial effusion in hypothyroidism is multifactorial: reduced thyroid hormone levels lead to increased capillary permeability, decreased albumin synthesis, and impaired lymphatic drainage, resulting in the accumulation of protein-rich serous fluid in the pericardial space [1,4,6]. The incidence of pericardial effusion in hypothyroid patients varies widely, reported in 3%-37% of cases, although clinically significant effusions are uncommon [4-6].

Similarly, hypothyroid myopathy is a well-recognized but often underdiagnosed manifestation, typically presenting with muscle stiffness, cramps, fatigue, and proximal weakness [7]. The underlying mechanisms include slowed muscle relaxation, impaired glycogenolysis, and mitochondrial dysfunction, leading to energy depletion and elevated serum creatine kinase (CK) levels [7,8]. In severe cases, this metabolic disturbance may progress to rhabdomyolysis, especially when compounded by other stressors such as infection, trauma, or medication use [7].

Due to the nonspecific and protean nature of these manifestations, recognition of severe hypothyroidism in such presentations requires a high index of suspicion [1,4]. In this report, we describe a case of newly diagnosed severe hypothyroidism presenting with hemodynamically significant bradycardia, moderate pericardial effusion, rhabdomyolysis, and bicytopenia, highlighting the diagnostic challenges and management strategies involved in such a multifaceted clinical scenario.

## Case presentation

History

A 45-year-old man with no known comorbidities presented to the emergency department with a three-day history of recurrent retrosternal, stabbing chest pain radiating to the left shoulder. The episodes, approximately 10 in total, lasted a few minutes each and resolved spontaneously, with no aggravating or relieving factors. He also reported persistent generalized weakness, especially on exertion, consistent with proximal muscle weakness. He denied weight changes, changes in appetite, cold intolerance, sleep disturbances, constipation or diarrhea, dyspnea, orthopnea, palpitations, syncope, peripheral edema, hair loss, skin changes, or facial swelling. He had two episodes of nonbilious, nonbloody vomiting in the 24 hours before presentation. His past medical history was notable for anemia diagnosed a few years earlier without further evaluation. He was a nonsmoker, did not consume alcohol, and worked as a gardener. There was no family history of thyroid disease or autoimmune disorders.

Examination

On physical examination, the patient was alert and oriented. Vital signs were temperature 36.8°C, heart rate 43 bpm, blood pressure 90/57 mmHg, respiratory rate 23 breaths/minute, and SpO₂ 96% on room air. He had bradycardia with muffled heart sounds, but no murmurs, rubs, or gallops were appreciated. Jugular venous distension was absent, and there were no signs of peripheral edema. Lung examination revealed clear breath sounds bilaterally without rales or wheezing. Abdominal examination was unremarkable, with no tenderness, organomegaly, or ascites. Neurological examination demonstrated normal cranial nerves, normal tone, and reflexes. Endocrine examination revealed mild proximal muscle weakness in the upper and lower limbs. There was no tremor, diaphoresis, exophthalmos, goiter, thyroid enlargement, lymphadenopathy, dry skin, hair thinning, or myxedematous features, and deep tendon reflexes were normal with no delayed relaxation. Skin and mucous membranes were otherwise normal.

Management

**Figure 1:**
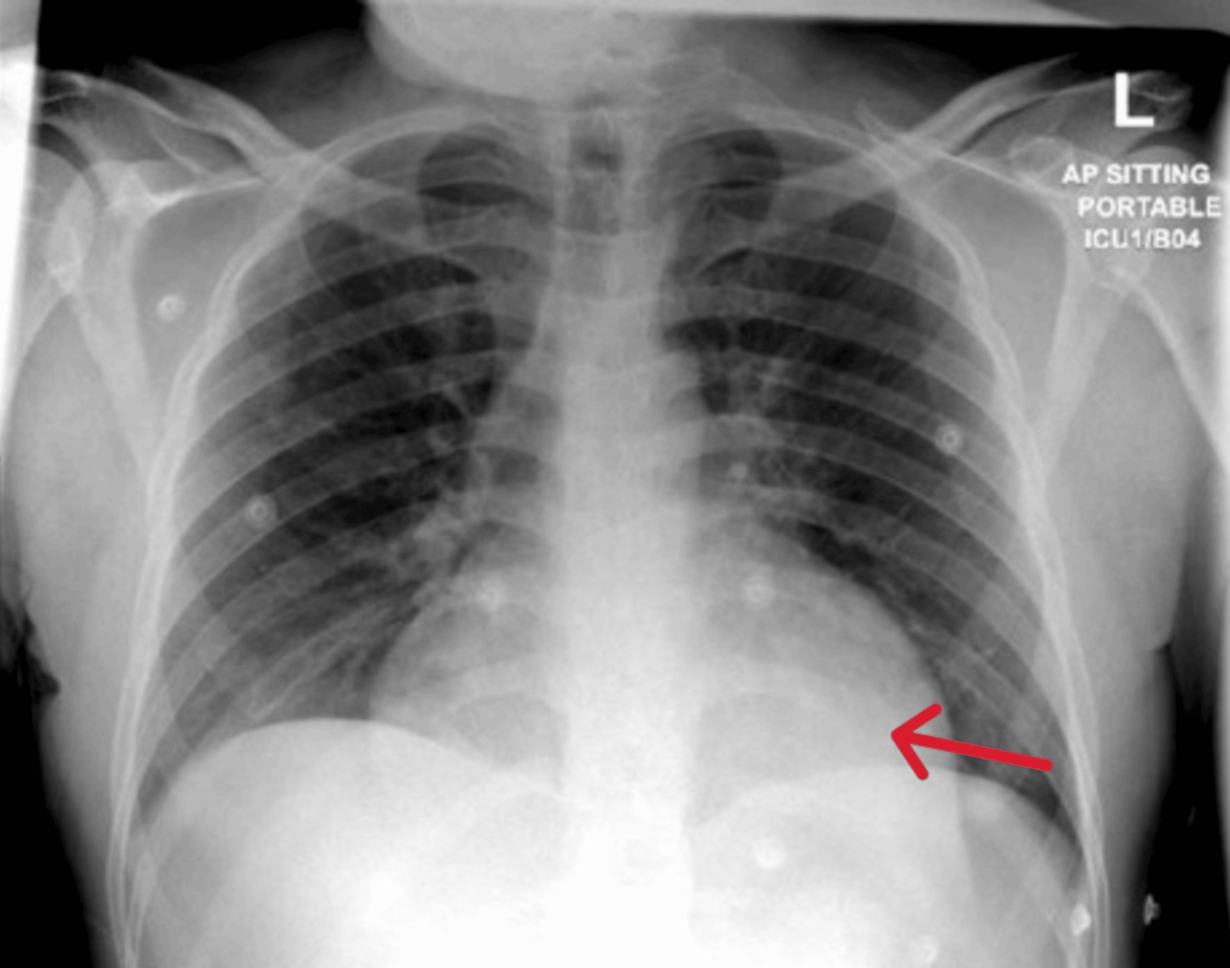
Chest X-ray, posteroanterior view, showing marked enlargement of the cardiac silhouette consistent with cardiomegaly (red arrow)

Initial laboratory investigations revealed normocytic normochromic anemia, mild thrombocytopenia, elevated creatinine, mild transaminitis, significantly elevated troponin, markedly elevated CK, and slightly elevated myoglobin (Table 1). Chest X-ray showed cardiomegaly suggestive of pericardial effusion (Figure 1).

**Table 1:** Laboratory parameters on admission and at discharge WBC: white blood cell; Hb: hemoglobin; Hct: hematocrit; MCV: mean corpuscular volume; MCH: mean corpuscular hemoglobin; Plt: platelets; CRP: C-reactive protein; Fe: iron; TIBC: total iron-binding capacity; Tf: transferrin; Fe sat.: iron saturation; Urea: serum urea; Cr: creatinine; Na⁺: sodium; K⁺: potassium; Ca²⁺: calcium; Mg²⁺: magnesium; TP: total protein; Alb: albumin; ALP: alkaline phosphatase; ALT: alanine aminotransferase; AST: aspartate aminotransferase; T. Bil: total bilirubin; HbA1c: hemoglobin A1c; TSH: thyroid-stimulating hormone; FT4: free thyroxine; PT: prothrombin time; INR: international normalized ratio; APTT: activated partial thromboplastin time; NT-proBNP: N-terminal pro-B-type natriuretic peptide; CK: creatine kinase; Mb: myoglobin; TnT: troponin T

Parameters	On admission	On discharge	Reference values (with units)
WBC	4.1	3.9	6.2 × 10³/µL
Hb	8.7	10.2	13-17 g/dL
Hct	26.1	33.4	40%-50%
MCV	90.9	98.2	83-101 fL
MCH	30.3	30.0	27-32 pg
Plt	142	210	150-410 × 10³/µL
CRP	2.2	-	0-5 mg/L
Fe	8	-	6-35 µmol/L
TIBC	60	-	45-80 µmol/L
Tf	2.4	-	2.0-3.6 g/L
Fe sat.	13	-	15%-45%
Urea	7.4	6.0	2.5-7.8 mmol/L
Cr	172	83	62-106 µmol/L
Na⁺	135	143	133-146 mmol/L
K⁺	3.9	4.8	3.5-5.3 mmol/L
Ca²⁺	2.22	-	2.2-2.6 mmol/L
Mg²⁺	0.89	-	0.7-1.0 mmol/L
pH	7.37	-	7.32-7.42
TP	77	75	60-80 g/L
Alb	41	39	35-50 g/L
ALP	44	60	40-129 U/L
ALT	33	23	0-41 IU/L
AST	106	23	0-41 IU/L
T. Bil	5	6	0-21 µmol/L
HbA1c	5.5	-	<6%
TSH	>100	22.2	0.34-4.20 mIU/L
FT4	<0.5	12.3	11-23.3 pmol/L
PT	11.6	-	9.4-12.5 seconds
INR	1.0	-	<1.0
APTT	28.4	-	25.1-36.5 seconds
NT-proBNP	145	-	<300 pg/mL
CK	5,173	3,757	39-308 U/L
Mb	169	104	28-72 ng/mL
TnT	201	114	3-15 ng/L

**Figure 2:**
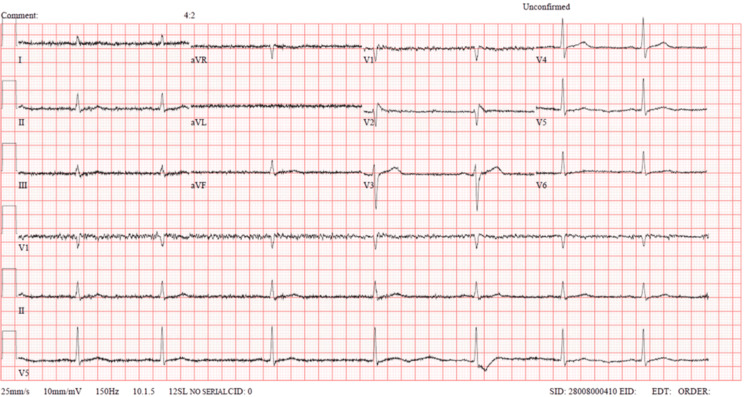
Electrocardiography revealing sinus bradycardia with a heart rate of 43-45 bpm. There were no ST-segment elevations or depressions, T-wave inversions, or pathological Q waves aVR: augmented vector right; aVL: augmented vector left; aVF: augmented vector foot

The patient was admitted as a possible case of non-ST elevation myocardial infarction (NSTEMI) and acute kidney injury secondary to rhabdomyolysis. Heart rate remained persistently low (39-49 bpm) (Figure 2) with borderline hypotension.

Transthoracic echocardiography revealed a normal ejection fraction (53%) and moderate pericardial effusion without tamponade (Video 1).

**Video 1:** Transthoracic echocardiography revealed an anechoic space surrounding the heart, consistent with moderate pericardial effusion. There were no echocardiographic signs of cardiac tamponade, such as diastolic right ventricular collapse or an enlarged inferior vena cava without respiratory variation

Further evaluation was undertaken due to unexplained bradycardia and hypotension. Thyroid function testing revealed severe primary hypothyroidism: thyroid-stimulating hormone (TSH) >100 mIU/L and free thyroxine (FT4) <0.4 ng/dL. Cortisol levels were at the lower limit of normal, but the short Synacthen (adrenocorticotropic hormone stimulation) test was normal (Table 1).

The diagnosis was revised to newly onset severe hypothyroidism presenting with bradycardia, hypotension, and pericarditis with moderate pericardial effusion. The patient was started on levothyroxine 75 μg daily, titrated to 125 μg daily, with gradual improvement in heart rate and clinical status (we initiated levothyroxine at a low dose (75 μg), taking into account the possibility of NSTEMI on admission, and gradually increased the dose to 125 μg as the patient tolerated therapy over a period of six days). Antithyroid peroxidase and antithyroglobulin antibodies were negative.

He was concurrently treated with aspirin 100 mg daily, clopidogrel 75 mg daily, atorvastatin 40 mg daily, and colchicine 0.5 mg twice daily. Follow-up echocardiography showed a stable pericardial effusion without hemodynamic compromise or signs of tamponade. The aspirin, clopidogrel, and atorvastatin were initially started due to the patient’s chest pain and elevated troponin, raising suspicion for NSTEMI. These were discontinued once ischemia was ruled out, while colchicine was given for pericardial inflammation. Cardiology recommended outpatient follow-up, expecting gradual resolution of the effusion over the next two to three months.

The patient was discharged in stable condition with the final diagnosis of severe hypothyroidism presenting with bradycardia, pericardial effusion, possible NSTEMI versus pericarditis-related troponin elevation, rhabdomyolysis, and bicytopenia likely secondary to hypothyroidism. At two-week follow-up, he was clinically very well and scheduled for further cardiology assessment, including consideration of elective coronary angiography and repeat echocardiography.

## Discussion

Severe hypothyroidism exerts significant effects on the cardiovascular system, many of which can mimic or complicate primary cardiac diseases, making diagnosis challenging. Reduced thyroid hormone levels lead to decreased myocardial contractility, heart rate, and cardiac output, contributing to bradycardia and diastolic dysfunction, as seen in our patient. These effects are mediated by impaired gene expression of calcium-handling proteins and downregulation of beta-adrenergic receptors, resulting in both decreased chronotropy and inotropy [1,2]. The reduced cardiac output, combined with increased systemic vascular resistance, often results in borderline hypotension and can precipitate heart failure if left untreated [3]. Our patient's presentation with bradycardia and moderate pericardial effusion is consistent with the known cardiovascular manifestations of overt hypothyroidism, although cardiac tamponade remains a rare complication [5,6]. The combination of moderate pericardial effusion, rhabdomyolysis, and bicytopenia observed in our patient is exceptionally rare, highlighting the novelty and clinical significance of this case.

Pericardial effusion is a well-documented but often underrecognized complication of hypothyroidism, resulting from increased capillary permeability and impaired lymphatic drainage, which lead to the accumulation of protein-rich fluid in the pericardial space [1,4]. While most pericardial effusions in hypothyroid patients are hemodynamically insignificant, large effusions causing tamponade are uncommon but potentially life-threatening [5,6]. Echocardiographic assessment should be structured to differentiate between global or localized effusion, quantify the effusion, describe fluid appearance, and analyze hemodynamic compromise. Standard views, along with two-dimensional echocardiography, M-mode, and Doppler analysis, should be used to ensure accurate evaluation of pericardial involvement [8]. In our case, moderate pericardial effusion without tamponade features highlights the importance of timely cardiac imaging in hypothyroid patients presenting with chest pain or hemodynamic changes.

Elevated cardiac biomarkers add diagnostic complexity. While elevated troponin typically suggests ischemic injury, in hypothyroidism, it may reflect secondary myocardial stress rather than ischemic injury [1,5]. Rhabdomyolysis, indicated by elevated CK and myoglobin, is a rare but recognized muscular manifestation of severe hypothyroidism, arising from metabolic disturbances that impair muscle membrane stability and mitochondrial function [7]. The concurrent presence of rhabdomyolysis and bicytopenia further illustrates the systemic impact of hypothyroidism, which may contribute to anemia and thrombocytopenia through bone marrow suppression and reduced erythropoiesis [1].

Hypothyroidism is associated with a spectrum of cardiovascular manifestations, including exercise intolerance, dyspnea, bradycardia, diastolic hypertension, and edema. Patients at higher risk for progression to myxedema coma generally include those with prolonged untreated hypothyroidism, advanced age, infections, trauma, or cold exposure, with reported mortality remaining high [9]. Our patient remained alert, normothermic, and hemodynamically stable, supporting a diagnosis of noncomatose severe hypothyroidism amenable to gradual levothyroxine replacement [1,7]. Patients at greater risk for progression to myxedema coma include older adults, individuals with cardiovascular disease, those with infections or trauma, and patients with prolonged untreated hypothyroidism. Early recognition and treatment are essential, as factors such as hypothermia, bradycardia, hypotension, and altered mental status are associated with myxedema coma, which, although rare, carries a high mortality if not promptly managed [10].

Levothyroxine remains the cornerstone of therapy for reversing cardiovascular and systemic manifestations of hypothyroidism [11]. In patients with cardiac involvement, including bradycardia and pericardial effusion, gradual dose escalation is recommended (typically starting 25-75 µg daily and titrating every one to two weeks) to minimize the risk of precipitating myocardial ischemia or arrhythmias [12]. The adjunctive use of anti-inflammatory agents, such as colchicine, employed here, aligns with current guidelines for pericarditis management [5]. Initial empiric therapy with aspirin, clopidogrel, and atorvastatin was started due to the suspicion of NSTEMI but discontinued once ischemia was ruled out.

Diagnostic and monitoring markers for hypothyroidism-related cardiac effects include standard thyroid function tests (TSH, FT4, FT3); electrocardiography to detect bradycardia or conduction abnormalities; echocardiography to evaluate pericardial effusion and ventricular function; advanced echocardiography, such as speckle-tracking, for subtle myocardial dysfunction; and biomarkers of cardiac stress, including troponin and NT-proBNP.

This case highlights the diagnostic complexity of severe hypothyroidism, which may present with atypical multisystem complications. The rare constellation of pericardial effusion, rhabdomyolysis, and bicytopenia underscores the importance of a comprehensive clinical, laboratory, and imaging evaluation. Timely recognition and management can prevent progression to life-threatening complications, avoid unnecessary interventions, and result in excellent clinical outcomes.

## Conclusions

Severe hypothyroidism may present with atypical and multisystem manifestations that mimic acute cardiac or metabolic emergencies. Early recognition and appropriate thyroid hormone replacement are vital to reversing potentially serious complications such as pericardial effusion, bradycardia, and rhabdomyolysis. Clinician awareness of these unusual presentations is key to timely diagnosis and improved patient outcomes.
